# Plasma Chemokine signature correlates with lung goblet cell hyperplasia in smokers with and without chronic obstructive pulmonary disease

**DOI:** 10.1186/s12890-015-0103-2

**Published:** 2015-09-30

**Authors:** Victor Kim, William D. Cornwell, Michelle Oros, Heba Durra, Gerard J. Criner, Thomas J. Rogers

**Affiliations:** Division of Pulmonary and Critical Care Medicine, Temple University School of Medicine, 3401 North Broad Street, 785 Parkinson Pavilion, Philadelphia, PA 19140 USA; Center for Inflammation, Translational and Clinical Lung Research, Temple University School of Medicine, Philadelphia, PA USA; Department of Pathology, Temple University School of Medicine, Philadelphia, PA USA

**Keywords:** Mucin, Chronic obstructive pulmonary disease, Goblet cell hyperplasia, Chemokine, Neutrophil, Macrophage

## Abstract

**Background:**

Chronic Obstructive Pulmonary Disease (COPD) is characterized by lung and systemic inflammation as well as airway goblet cell hyperplasia (GCH). Mucin production is activated in part by stimulation of the epidermal growth factor (EGF) receptor pathway through neutrophils and macrophages. How circulating cytokine levels relate to GCH is not clear.

**Methods:**

We performed phlebotomy and bronchoscopy on 25 subjects (six nonsmokers, 11 healthy smokers, and eight COPD subjects FEV_1_ 30–60 %). Six endobronchial biopsies per subject were performed. GCH was measured by measuring mucin volume density (MVD) using stereological techniques on periodic acid fast-Schiff stained samples. We measured the levels of chemokines CXCL8/IL-8, CCL2/MCP-1, CCL7/MCP-3, CCL22/MCD, CCL3/MIP-1α, and CCL4/MIP-1β, and the cytokines IL-1, IL-4, IL-6, IL-9, IL-17, EGF, and vascular endothelial growth factor (VEGF). Differences between groups were assessed using one-way ANOVA, *t* test, or Chi squared test. Post hoc tests after ANOVA were performed using Bonferroni correction.

**Results:**

MVD was highest in healthy smokers (27.78 ± 10.24 μL/mm^2^) compared to COPD subjects (16.82 ± 16.29 μL/mm^2^, *p* = 0.216) and nonsmokers (3.42 ± 3.07 μL/mm^2^, *p* <0.0001). Plasma CXCL8 was highest in healthy smokers (11.05 ± 8.92 pg/mL) compared to nonsmokers (1.20 ± 21.92 pg/mL, *p* = 0.047) and COPD subjects (6.01 ± 5.90 pg/mL, *p* = 0.366). CCL22 and CCL4 followed the same trends. There were no significant differences in the other cytokines measured. When the subjects were divided into current smokers (healthy smokers and COPD current smokers) and non/ex-smokers (nonsmokers and COPD ex-smokers), plasma CXCL8, CCL22, CCL4, and MVD were greater in current smokers. No differences in other cytokines were seen. Plasma CXCL8 moderately correlated with MVD (*r* = 0.552, *p* = 0.003).

**Discussion:**

In this small cohort, circulating levels of the chemokines CXCL8, CCL4, and CCL22, as well as MVD, attain the highest levels in healthy smokers compared to nonsmokers and COPD subjects. These findings seem to be driven by current smoking and are independent of airflow obstruction.

**Conclusions:**

These data suggest that smoking upregulates a systemic pattern of neutrophil and macrophage chemoattractant expression, and this correlates significantly with the development of goblet cell hyperplasia.

## Background

Chronic Obstructive Pulmonary Disease (COPD) is characterized by an abnormal inflammatory response to noxious environmental stimuli in the lung [1]. Persistent lung inflammation leads to the development of emphysema and airway disease, of which goblet cell hyperplasia (GCH) is a crucial component [2]. GCH is a common phenomenon in COPD, regardless of the presence or absence of chronic bronchitis (CB) [3–5]. It has been shown that subjects with more airflow obstruction have a greater burden of mucus in the small airways [4]. In addition, a bronchoscopic study in smokers with and without airflow obstruction demonstrated more GCH in the large airways, particularly in those with COPD [5]. However, there is a large disconnect between symptoms of cough and sputum and mucus burden [6]. The most well characterized pathologic index described by Reid has shown only a weak relationship between chronic bronchitic symptoms [7].

While well described in asthma, the inflammatory mechanisms responsible for GCH in COPD are not well known. It has been established that subjects with airflow obstruction demonstrate greater neutrophilic, lymphocytic, and macrophage infiltration in the lung parenchyma which increases as lung function declines [3, 4, 8], but how trafficking of these cells to the airways occurs is not known. To complicate matters, it has been increasingly recognized that COPD is associated with significant systemic inflammation, but the association between systemic inflammation and lung GCH is not known. We sought to characterize the systemic cytokine profiles and relate them to GCH. We hypothesized that elevations in systemic cytokines would be associated with increased GCH as a result of immune trafficking to the lung and subsequent mucin production. Specifically, because neutrophils and macrophages are associated with mucin gene expression [9], we hypothesized that plasma proinflammatory chemokines which mobilize neutrophils and macrophages, particularly interleukin-8 (CXCL8), monocyte chemotactic proteins-1 and −3 (CCL2 and CCL7), macrophage derived chemokine (CCL22), and macrophage inflammatory proteins-1α and -1β (CCL3 and CCL4), would correlate with GCH in smokers and ex-smokers with and without COPD.

## Methods

This study was approved by the Temple University School of Medicine IRB (protocol no. 20687). Written informed consent to participate was obtained by the PI (VK). We performed phlebotomy and bronchoscopy on 25 subjects (six nonsmokers, 11 healthy smokers, and eight COPD subjects). Inclusion and exclusion criteria are summarized in Table 1. Briefly, for COPD subjects, we included those with an FEV_1_ between 30 and 60 %, because this group is considered at high risk for exacerbation. Healthy smokers needed to have at least a 10-pack year history of smoking. Healthy nonsmokers served as a control group. We excluded those with upper airway disease such as allergic rhinitis or chronic sinusitis, those with a COPD exacerbation, upper respiratory tract infection, or acute sinusitis within 6 weeks prior to bronchoscopy in order to exclude the possible effects of upper airway GCH on lower airway GCH. We also excluded those with abnormal coagulation profile or on anticoagulation within 6 half-lives of the bronchoscopy, and those with a known allergy to lidocaine. Subjects treated with inhaled corticosteroids had a washout period of 4 weeks prior to bronchoscopy, to negate their possible effects on GCH. We excluded those deemed high risk for discontinuation of inhaled corticosteroids (e.g., history of frequent exacerbations).

**Table 1:** Inclusion and exclusion criteria

Inclusion criteria
Age between 40 and 70 years
Diagnosis of COPD or at risk for COPD
Smoking History >10 pack years (for nonsmokers less than 100 cigarettes in lifetime)
FEV_1_ 30–60 % (COPD group), normal FEV_1_ (Healthy Smoker Group)
English speaking
Exclusion criteria
Diagnosis of chronic sinusitis or allergic rhinitis
Presence of other lung disease (including asthma)
Pregnancy
Sinusitis or URI within the last 6 weeks
COPD Exacerbation within 6 weeks of screening visit
Presence of infiltrate or mass on CT scan
Anticoagulation or antiplatelet therapy within 6 half lives of bronchoscopy
Known allergy to lidocaine
Predisposition to bleeding
Chronic treatment with steroids, oral or inhaled that cannot be discontinued for 4 weeks prior to study
Unwillingness to participate in study

Six endobronchial biopsies per subject were performed. After premedication with intravenous fentanyl and midazolam, bronchoscopy was performed using local anesthesia with topical lidocaine. Endobronchial mucosal biopsies were performed at carinae of segmental airways, in the right lower lobe, right middle lobe, and right secondary carina (branch point between right upper lobe and bronchus intermedius). Plasma was collected on the same day as bronchoscopy. Briefly, 20 ml of blood was collected by venous puncture into vacutainers containing EDTA as the anticoagulant. The blood was layered on 15 ml of Ficoll-Paque Plus (GE Healthcare), and centrifuged for 40 min at 300 g. The plasma layer was recovered and stored at −80 °C.

**Fig. 1:**
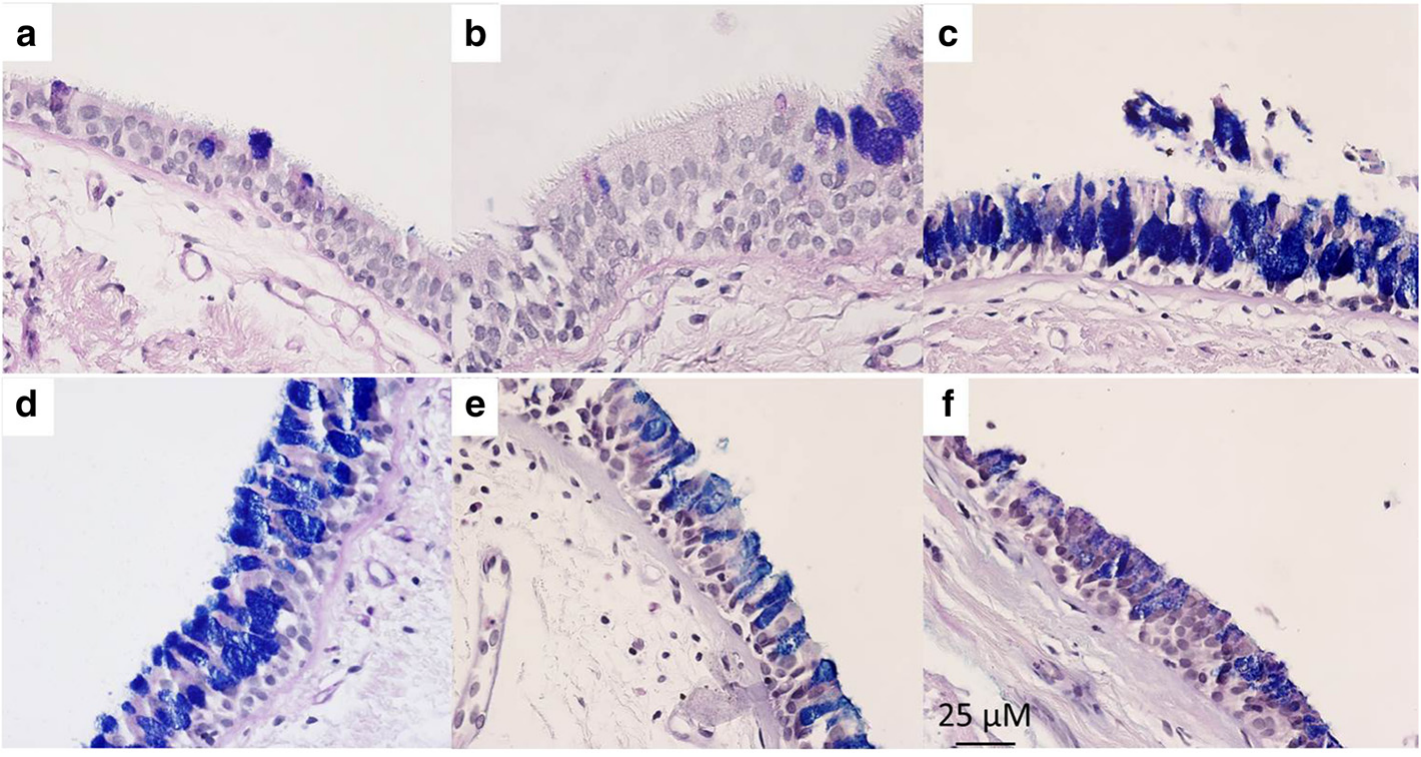
Examples of mucosal biopsies from (**a**) and (**b**) healthy nonsmokers, (**c**) and (**d**) healthy smokers,and (**e**) and (**f**) COPD subjects, taken at 40×. Specimens stained with periodic acid Schiff-Alcian Blue, staining goblet cells blue/purple

GCH was measured by measuring mucin volume density (MVD) using stereological techniques on periodic acid fast-Schiff stained samples. Examples of images from a healthy nonsmoker and a healthy smoker are shown in Fig. 1. Mucin volume was measured using a modified model described by us [10] using Image J. Length of basement membrane (L_BM_) and total area of mucin granules (MA) were measured. MVD (μL/mm^2^) was calculated using stereologic techniques as described previously [11, 12]: MVD = MA/(L_BM_)(4/π).

Plasma was analyzed for cytokine and chemokine levels using the Luminex platform. EMD Millipore cytokine kit, HCYTOMAG-60K-29, was purchased and the following analytes measured: IL-1β, IL-4, IL-6, CXCL8, IL-9, IL-12p40, IL-15, IL-17, CCL2, CCL7, CCL22, CCL3, CCL4, Eotaxin, IP-10, interferon-gamma (IFN-γ), granulocyte colony stimulating factor (G-CSF), epidermal growth factor (EGF), IFN-α2, transforming growth factor-alpha (TGF-α), and vascular endothelial growth factor (VEGF).

## Statistics

Statistical analysis was performed using SPSS v22 (SAS, Cary, NC) and graphs created with Graphpad Prism v6.03. Differences between the three groups (nonsmokers, healthy smokers, COPD) were assessed using one-way ANOVA or Chi squared test. Post hoc tests after ANOVA were performed using Bonferroni correction. In addition, an analysis of all current smokers vs. all non- or ex-smokers was performed with *t* test and Chi squared test. A *p* value of <0.05 was considered statistically significant. Correlations between plasma cytokines and MVD were performed using Spearman’s correlation.

## Results

The clinical and physiologic characteristics, as well as the MVD, of the subjects are summarized in Table 2. There was no statistically significant difference in age, gender, or body mass index between groups. There were more African-Americans in the COPD group compared to the healthy smoker and nonsmoker groups. By definition, the COPD group had worse spirometry compared to the healthy smoker and nonsmoker group, and smoking history was not different between the COPD and healthy smoker groups. Five out of eight (62.5 %) of the COPD group were current smokers, compared to 100 % in the healthy smoker group. Five out of eight (62.5 %) in the COPD group had chronic bronchitis, defined by chronic cough and sputum at least 3 months a year for at least two consecutive years. Two out of the 11 healthy smokers (18.2 %) had chronic bronchitis. To our surprise, the MVD was greatest in the healthy smoker group (27.78 ± 10.24 μL/mm^2^) compared to the nonsmoker group (3.42 ± 3.07 μL/mm^2^, *p* <0.001). In the COPD group, the MVD was less than the healthy smoker group (16.82 ± 16.29 μL/mm^2^), but the difference was not statistically significant (*p* = 0.216).

**Table 2:** Demographic factors, BAL results, mucin volume density

	Nonsmokers	Healthy smokers	COPD	
	*n* = 6	*n* = 11	*n* = 8	*p*
Age (years)	47.60 ± 11.82	49.58 ± 5.57	57.0 ± 4.66	0.052
Gender (male, n (%))	4 (66.7)	4 (36.3)	5 (62.5)	0.327
BMI (kg/m^2^)	31.13 ± 5.26	30.78 ± 4.74	29.30 ± 5.88	0.743
Race^a^ (White, n (%))	3 (50.0)	2 (18.2)	0 (0)	0.035
FEV_1_ ( % pred)	89.50 ± 20.52*	101.17 ± 16.30*	46.25 ± 8.83	<0.0001
FVC ( % pred)	92.90 ± 16.23	103.67 ± 16.87*	74.13 ± 12.70	0.001
FEV_1_/FVC	88.2 ± 21.70*	96.67 ± 6.14*	52.38 ± 11.95	<0.0001
Smoking History (pack years)	0* **	25.58 ± 10.66	22.25 ± 9.00	<0.0001
Current Smoking (n, %)	0 (0)	11 (100)	5 (62.5)	<0.0001
Chronic Bronchitis (n, %)	0 (0)	2 (18.2)	5 (62.5)	0.006
MVD (μL/mm^2^)	3.42 ± 3.07**	27.78 ± 10.24	16.82 ± 16.29	0.002

^a^Other race is Black. *p <0.05 compared to COPD, **p <0.05 compared to Healthy Smokers

**Fig. 2:**
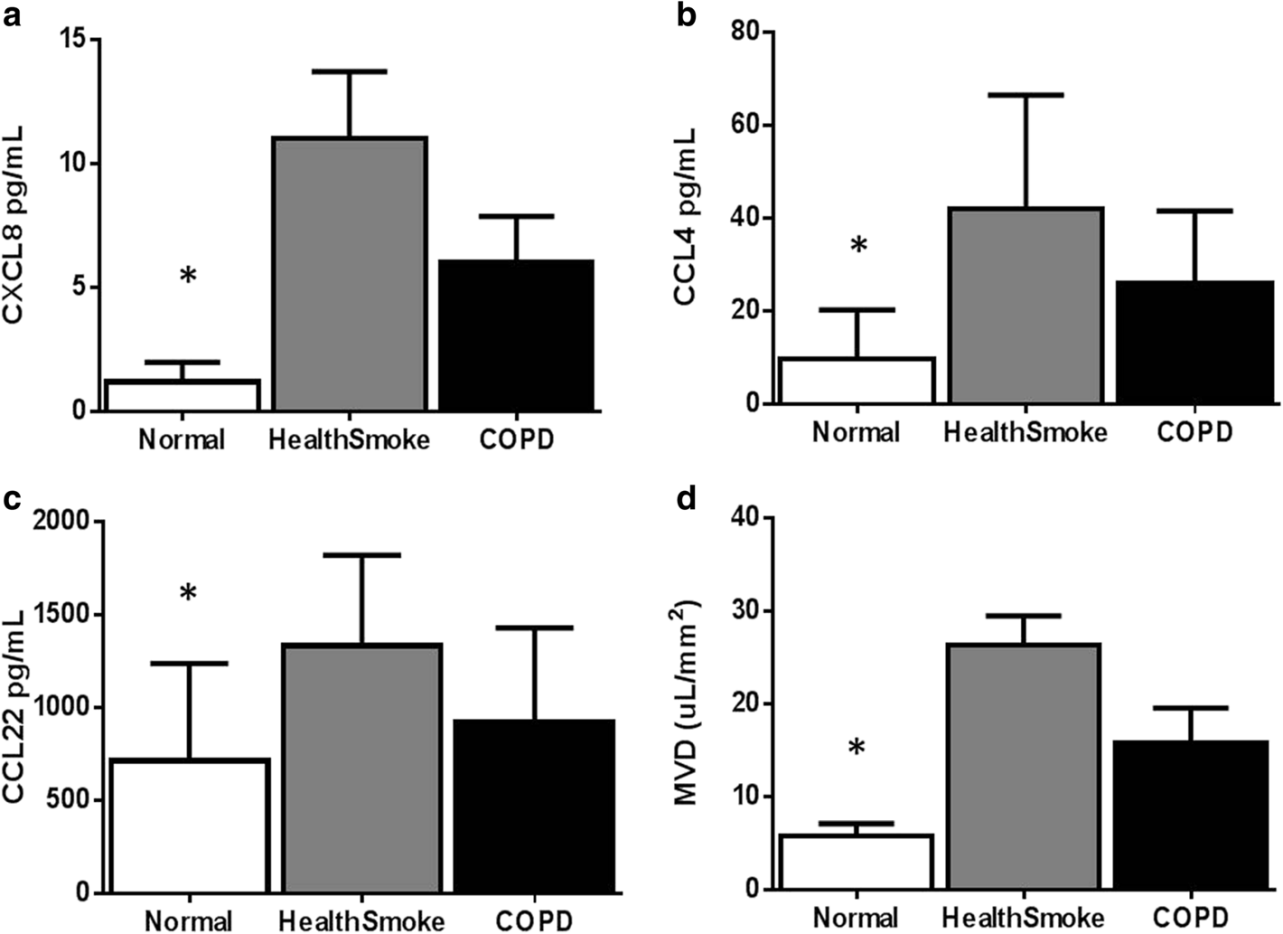
Plasma cytokines **a**) CXCL8, **b**) CCL4, **c**) CCL22 and **d**) MVD by group. *p <0.05 compared to healthy smokers

The levels of plasma chemokines and cytokines are summarized in Table 3. Plasma CXCL8 was greatest in the healthy smoker group (11.05 ± 8.92 pg/mL) compared to the nonsmoker group (1.20 ± 21.92 pg/mL, *p* = 0.047) and COPD group (6.01 ± 5.90 pg/mL, *p* = 0.366). See Fig. 2. Similar trends were seen in CCL22 and CCL4, where concentrations of these chemokines were greatest in the healthy smoker group, and significantly different from the nonsmoker group but not the COPD group. CCL7 was greatest in the COPD group (50.74 ± 25.88 pg/mL) compared to the nonsmoker group (17.33 ± 14.44 pg/mL, *p* = 0.028) and the healthy smoker group (40.66 ± 22.34 pg/mL, *p* = 0.167). There were no significant differences between groups in CCL2, CCL3, or other cytokines.

**Table 3:** Plasma chemokines

		Nonsmokers (*n* = 6)	Healthy smokers (*n* = 11)	COPD (*n* = 8)	*P*
Chemokines	Eotaxin	83.92 ± 57.41	96.16 ± 58.86	96.42 ± 75.40	0.920
	CXCL8	1.20 ± 21.92*	11.05 ± 8.92	6.01 ± 5.90	0.028
	IP10	233.94 ± 125.07	671.55 ± 544.81	670.36 ± 751.53	0.287
	CCL2	200.98 ± 128.03	310.63 ± 149.19	207.02 ± 76.06	0.106
	CCL7	17.33 ± 17.44**	40.66 ± 22.34	50.74 ± 25.88	0.031
	CCL22	714.23 ± 524.71*	1335.51 ± 486.74**	750.37 ± 251.39	0.009
	CCL3	17.44 ± 25.47	7.88 ± 21.72	8.33 ± 3.55	0.255
	CCL4	9.69 ± 10.50*	42.04 ± 24.48	25.98 ± 15.60	0.009
Th1	IFNγ	4.21 ± 2.63	9.58 ± 6.13	19.24 ± 28.76	0.253
	IL12p40	Undetectable	38.97 ± 8.01**	20.44 ± 17.22	0.035
Th2	IL4	10.71 ± 13.20	15.35 ± 12.26	47.77 ± 81.07	0.256
	IL9	1.16 ± 1.83	1.71 ± 1.44	2.66 ± 2.95	0.389
Th17	GCSF	83.92 ± 57.41	74.14 ± 81.80	25.79 ± 25.96	0.059
	IL6	11.39 ± 2.58	9.46 ± 2.46	64.27 ± 163.01	0.410
	IL17	Undetectable	8.76 ± 0.87	8.62 ± 1.03	0.809
Other	EGF	12.65 ± 3.31	13.96 ± 8.19	19.16 ± 19.66	0.559
	IFNα2	17.19 ± 11.28	57.43 ± 37.42	67.87 ± 71.16	0.155
	IL1β	2.86 ± 4.44	1.97 ± 2.59	5.30 ± 7.01	0.323
	IL10	9.35 ± 7.73	6.78 ± 6.08	16.11 ± 27.39	0.478
	IL15	5.18 ± 3.52	5.53 ± 3.20	5.75 ± 5.19	0.965
	TGFα	Undetectable	Undetectable	Undetectable	
	VEGF	130.03 ± 100.23	195.44 ± 55.90	258.95 ± 295.52	0.427

*p <0.05 vs. healthy smokers, **p <0.05 vs. COPD

**Fig. 3:**
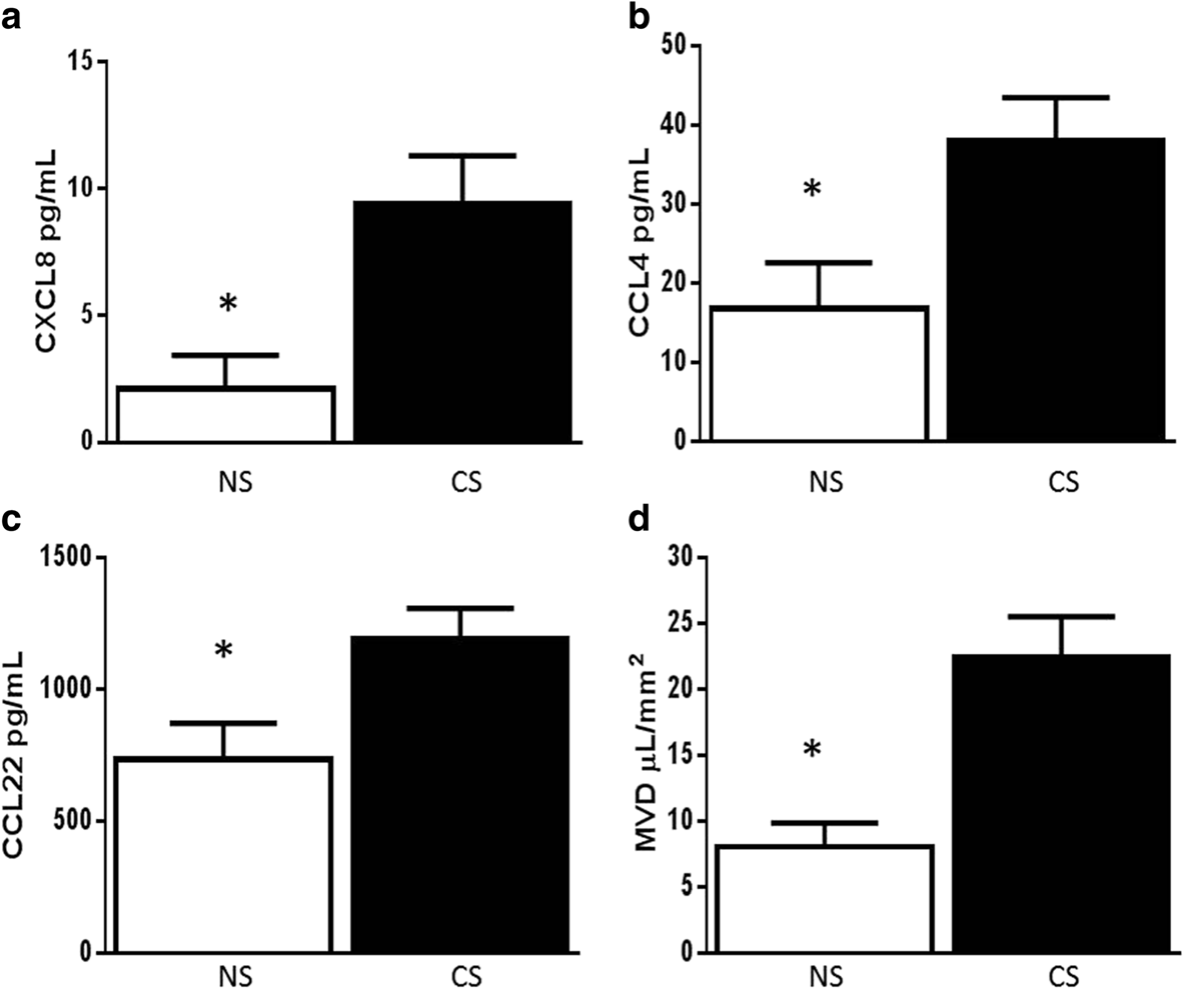
Plasma Cytokines **a**) CXCL8, **b**) CCL4, **c**) CCL22, and **d**) MVD by current smoking. *NS* Non- or ex-smokers, *CS* Current smokers. *p <0.05 compared to current smokers

**Fig. 4:**
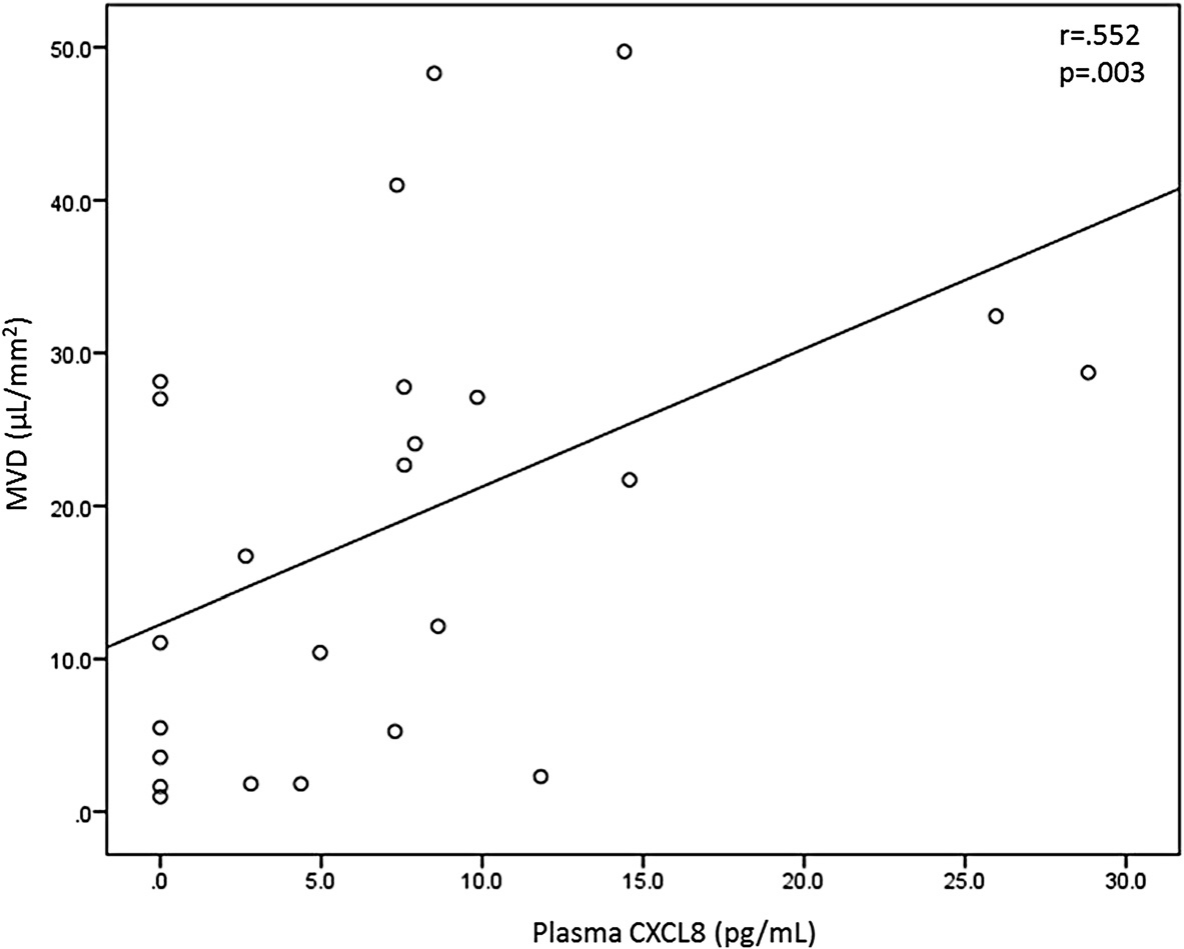
Relationship between mucus volume density and plasma CXCL8 concentrations

Subjects were subsequently divided into current smokers (including healthy smokers and COPD subjects) and non- or ex-smokers (nonsmoker group plus COPD subjects that quit smoking). As there were only three former smokers in the analysis, they were grouped together with the non-smokers. Plasma cytokine levels were compared between these two groups. The results are summarized in Table 4. The concentration of plasma CXCL8 was greater in the current smoker group compared to the non- or ex-smokers. Plasma CCL22 and CCL4 were also greater in the current smoker group. There were no significant differences in CCL2, CCL3, CCL7, or other cytokines between these groups. MVD was greater in the current smokers compared to the nonsmokers. See Fig. 3. Finally, there was a moderate correlation between plasma CXCL8 and MVD (*r* = 0.552, *p* = 0.003). See Fig. 4. There were no significant correlations with other plasma chemokines.

**Table 4:** Plasma chemokines and mucus volume density in current smokers vs. non- or ex-smokers

		Non- or ex-smokers (*n* = 9)	Current smokers (*n* = 16)	*p*
Chemokines	Eotaxin	88.87±84.90	95.87±51.47	0.791
	CXCL8	2.11±3.98	9.43±7.94	**0.016**
	IP10	295.19±157.43	713.20±676.25	0.082
	CCL2	213.03±113.31	265.32±135.19	0.329
	CCL7	36.33±34.82	40.65±20.23	0.686
	CCL22	653.56±439.54	1239.50±498.06	**0.006**
	CCL3	14.95±20.70	7.78±1.83	0.149
	CCL4	14.99±16.20	35.86±21.99	**0.019**
Th1	IFNγ	18.17±31.31	8.86±5.15	0.222
	IL12p40	30.15^a^	34.76±15.35	0.778
Th2	IL4	46.94±86.59	16.02±13.72	0.144
	IL9	2.43±3.42	1.70±1.30	0.426
Th17	GCSF	12.95±25.71	56.46±68.36	0.079
	IL6	71.61±171.16	9.48±3.39	0.129
	IL17	9.30^a^	8.64±0.93	0.510
Other	EGF	18.43±19.35	14.18±8.77	0.434
	IFNα2	46.90±77.84	55.08±35.52	0.708
	IL1β	5.47±7.02	2.37±3.57	0.137
	IL10	19.90±28.45	6.47±5.15	0.059
	IL15	6.89±5.07	4.86±3.23	0.217
	TGFα	Undetectable	Undetectable	
	VEGF	156.87±93.82	228.21±221.00	0.366
	MVD	7.41±7.56	24.07±13.70	**<0.0001**

Plasma chemokines expressed as pg/mL. MVD expressed as μL/mm^2^

^a^Only one sample with detectable levels

## Discussion

As previously shown, we found that GCH was greatest in smokers without airflow obstruction compared to COPD subjects and nonsmokers, and that this effect was primarily driven by current smoking [13]. In this small cohort, we demonstrated that plasma chemokines CXCL8, CCL22, and CCL4 were also greatest in the healthy smoker group, following the same pattern as the degree of GCH. These cytokines were also greater in those currently smoking, suggesting a causal relationship between smoking and these cytokines which result in GCH. Interestingly, we found that CCL7 was greatest in the COPD group, but found no other significant differences in other cytokines between groups. Finally, we showed that there was a moderate correlation with plasma CXCL8 concentrations with MVD. These findings suggest that smoking upregulates these plasma neutrophil and macrophage chemokines which result in the development of lung GCH.

Several cytokines and biomarkers have been examined in COPD subjects in various compartments, including plasma, sputum, and BAL. The most well characterized ones include C-reactive protein, fibrinogen, surfactant protein-D, IL-6, TNF-α, and CXCL8 [14–17]. However, prior literature has emphasized levels in COPD subjects in the chronic stable state compared to controls, compared to periods of exacerbation, or in response to therapy [14, 15, 17–19]. Hurst et al. found that systemic levels of IL-6 correlated with sputum concentrations of CXCL8 during exacerbations compared to the chronic stable state [15]. Sin et al. reported that inhaled fluticasone and fluticasone/salmeterol combination reduced systemic levels of surfactant protein-D but not C-reactive protein or IL-6 [19]. Few studies have addressed the role of cytokines in relationship to GCH in smokers with and without airflow obstruction. Interestingly, we found that the levels of certain chemokines, particularly CXCL8, were significantly correlated with GCH. Another interesting finding was that smokers without airflow obstruction had greater MVD compared to COPD subjects, a novel finding compared to prior studies [4, 5].

CXCL8 has been the subject of many prior investigations in COPD. CXCL8 is a potent neutrophil chemoattractant [20], which is a known stimulant of mucin production and degranulation of mucin stores [21]. CXCL8 also regulates mucin gene expression at the posttranscriptional level [22]. A bronchoscopic study of 39 COPD subjects and 18 healthy controls found that CXCL8 in BAL was significantly higher in frequent exacerbators [23]. Furthermore, recent studies have shown that CXCL8 levels are significantly elevated in the blood in COPD patients, and that CXCL8 (as well as CCL4) levels correlate with mortality, exacerbation rate, and BODE scores, and inversely correlate with FEV_1_ and DLCO [24–26]. Moreover, analysis of the levels of CXCL8 in BAL and sputum found higher levels of sputum CXCL8 in COPD subjects compared to healthy smokers and nonsmoking controls but no difference in BAL CXCL8 [27]. However, one study found BAL CXCL8 levels to be greater in smokers and COPD subjects compared to normal controls [28], and another study showed that BAL CXCL8 levels could distinguish current smokers with emphysema from those without emphysema [29]. In contradistinction to prior studies, we found that plasma CXCL8 levels to be highest in the healthy smoker group and was highly dependent on current smoking. Moreover, We found a moderate correlation between plasma CXCL8 and MVD, a novel finding in comparison with current literature, and as the greatest levels of each were found in current smokers, this association suggests a relationship with plasma neutrophils and the development of GCH in the lung. It is known that cigarette smoke causes influx of neutrophils and macrophages to the lung. Cigarette smoke extract has been shown to increase CXCL8 release from bronchial epithelial cells in a concentration- and time-dependent manner [30]. Cigarette smoking acutely increases plasma neutrophil activation as well in young smokers susceptible to the development of COPD (defined as those with familial aggregation) [31]. Our findings support this phenomenon by demonstrating the upregulation of the plasma levels of the chemokines CXCL8, CCL4, and CCL22 in current smokers.

Some studies have tried to relate cytokines with the presence of chronic bronchitis. A bronchoscopic study of 42 subjects with chronic bronchitis (with and without airflow obstruction) and 13 healthy controls found increased activity of CXCL8, myeloperoxidase, hyaluronan, and eosinophil cationic protein [32]. Sputum CCL2 levels have recently been shown to be increased in COPD subjects with chronic bronchitis compared to COPD subjects without chronic bronchitis [33]. In this study, sputum neutrophil and eosinophil counts were also higher in the chronic bronchitic group. Moreover, Monzon et al. described a CCL2/CCR2B dependent loop which appeared to upregulate mucin gene expression in human airway epithelial cells [34]. In contrast, de Moraes et al. found an association with serum IL-6 levels in ex-smoker COPD subjects with chronic bronchitis, but were unable to demonstrate a relationship of IL-6 or CXCL8 with disease severity [35].

There is growing evidence that CCL22 may be involved in the pathogenesis of chronic lung inflammation. CCL22 is produced predominantly by monocytes, macrophages, and dendritic cells, and is a potent chemoattractant for macrophages, NK cells, and some T cells [36]. It is apparent that the levels of CCL22 mRNA and protein are elevated in both lung tissue and lavage fluid in COPD [37]. The levels of CCL22 are also elevated in the lavage fluid obtained from patients with idiopathic pulmonary fibrosis [38]. Furthermore, Frankenberger et al. recently reported a 10-fold increase in the expression of CCL22 by macrophages obtained from BAL of either COPD patients or smokers [39]. Finally, Belperio et al. have shown that the levels of CCL22 are overexpressed in a bleomycin model of murine pulmonary fibrosis [40]. Similarly, the levels of this chemokine are significantly increased in the lungs in studies of a rat model of radiation pneumonitis-related pulmonary fibrosis [41].

Recent reports using experimental animal models have suggested that CCL4 may play an important role in the induction of lung inflammation following exposure to tobacco smoke. Ma et al. report in increase in production of CCL4 in the lungs of mice subjected to daily administration of cigarette smoke [42]. The receptor for this chemokine is CCR5, and the administration of tobacco smoke to CCR5-deficient mice shows substantially attenuated lung inflammation when compared to mice which express normal levels of CCR5 [42, 43]. These results suggest that the CCL4-CCR5 response loop may make a substantial contribution to the development of lung inflammation associated with smoke exposure. However, it should be pointed out that Meuronen et al. have reported that CCL4 levels are significantly decreased in the BAL of asymptomatic long-term smokers [44]. This report is in contrast to the results reported herein, or with the results of studies with chronic bronchitis patients in which the levels of CCL4 were found to be increased in lavage fluid [45]. An explanation for the disagreement in the results from these studies is not clear at this time. Less is known about CCL7 in COPD, but it has been described in other inflammatory diseases including asthma, multiple sclerosis, and rheumatoid arthritis [46]. We found that CCL7 plasma levels were greatest in the COPD group. Further study in a larger cohort is needed to validate these findings.

In the present study, some cytokines, and GCH, are downregulated in COPD patients compared to healthy smokers. Other studies have found that proinflammatory cytokines and GCH are upregulated in COPD [4, 5, 14]. Corticosteroids were withheld for four weeks prior to phlebotomy and bronchoscopy, so it is doubtful that the prior use of inhaled corticosteroids is responsible. We suggest that current smoking has a more powerful influence on circulating cytokine levels in this cohort.

This study has several limitations. Firstly, the sample size is small, meaning studies of greater magnitude are needed in order to confirm these findings. The biopsies are of the large airways, which may not represent disease of the smaller airways where airflow obstruction is thought to occur. Sampling error of the mucosa is a potential issue, but the biopsies were performed in systematic fashion and therefore less likely to be the cause of the findings. There were significant differences in racial distribution between the 3 groups, which may have had bearing on the results. By design, the study recruited those with moderate to severe COPD, so little can be said about those with milder disease. Finally, our data suggest that plasma levels of four chemokines statistically correlate with COPD, and three of these (CXCL8, CCL4 and CCL22) also correlate with mucous volume density. Nevertheless, at this point little can be said about immune trafficking of inflammatory cells into the lung, as plasma cytokine levels and GCH were measured separately and are purely associations at this point. Additional studies will be necessary to more fully assess the contribution of chemokine-driven inflammatory cell recruitment to the degree of lung disease, particularly mucous production, in COPD.

## Conclusions

We found greater degrees of GCH in the healthy smoking group and all current smokers, which correlated with differences in plasma CXCL8, CCL22, and CCL4 between groups in similar fashions. These associations suggest that smoking has a systemic effect on circulating cytokine levels that lead to the development of GCH. Further study is needed to corroborate these findings in a larger cohort.

## Abbreviations

ANOVA: Analysis of variance
BAL: Bronchoalveolar lavage
CB: Chronic bronchitis
COPD: Chronic obstructive pulmonary disease
CCL2: Monocyte chemotactic protein-1
CCL3: Macrophage inflammatory proteins-1α
CCL4: Macrophage inflammatory proteins-1β
CCL7: Monocyte chemotactic protein-3
CCL22: Macrophage derived chemokine
CXCL8: Interleukin 8
DLCO: Diffusing capacity of carbon monoxide
EGF: Epidermal growth factor
FEV_1_: Forced expiratory volume in 1 s
G-CSF: Granulocyte colony stimulating factor
GCH: Goblet cell hyperplasia
IFN-α2: Interferon-alpha2
IFN-γ: Interferon-gamma
L_BM_: Length of basement membrane
MA: Total area of mucin granules
MVD: Mucin volume density
TGF-α: Transforming growth factor-alpha
VEGF: Vascular endothelial growth factor
